# Additive Manufacturing of Dense Ti6Al4V Layer via Picosecond Pulse Laser

**DOI:** 10.3390/ma16010324

**Published:** 2022-12-29

**Authors:** Xiaomeng Zhu, Teng Yin, Yuzhou Hu, Siyuan Li, Dong Wu, Zhilin Xia

**Affiliations:** 1 School of Materials Science and Engineering, Wuhan University of Technology, Wuhan 430070, China; 2 State Key Laboratory of Silicate Materials for Architectures, Wuhan University of Technology, Wuhan 430070, China

**Keywords:** laser additive manufacturing, picosecond pulse laser, heat effect, nano–indentation, molten pool formation, laser-matter interaction

## Abstract

Ultrashort pulse laser shows good potential for heat control improvement in metal additive manufacturing. The challenge of applying ultrashort pulse laser as the heat source is to form a fully melted and dense microstructure. In this study, a picosecond pulse laser is introduced for fabricating single layer Ti6Al4V samples. The results, by examining through X-ray computed tomography (X-CT), scanning electron microscopy (SEM), show that highly dense Ti6Al4V samples were fabricated with optimized process parameters. The analysis of the cross section presents a three-zones structure from top to bottom in the sequence of the fully melted zone, the partially melted zone, and the heat-affected zone. A semi-quantitative study is performed to estimate the thermal efficiency of melted pool formation. The mechanical properties of the samples are tested using nano-indentation, showing an elastic modulus of 89.74 ± 0.74 GPa. The evidence of dense melted pool with good mechanical properties indicates that the picosecond laser can be integrated as the heat source with the current metal additive manufacturing to fabricate parts with accuracy control for the smaller size of thermal filed.

## 1. Introduction

Laser additive manufacturing (AM) processes, such as powder bed fusion laser (PBF-L) and directed energy deposition laser (DED-L), have great advantages in the manufacturing of metallic parts directly with complex structures because of their dimensional accuracy and excellent mechanical properties [1]. Since the issue of laser–matter interaction at the mesoscopic scale remains unclear, as reported by Gu [2], the optimization has limited scientific guidelines and is mainly empirical. In this situation, the input power of the laser is always considered as the primary factor to ensure dense and reliable fabrication. The suggested power of the continuous laser commonly ranges from 100 W to 1000 W for AM of metallic parts. High laser power brings about the quality of products, and the excess energy input also raises the challenge for heat control [3,4]. The approach for improving heat control issue is using pulse laser. Millisecond laser was used to print single tracks and layers of 316 L stainless steel in SLM, trying to provide better control of the heat input and solidification kinetics [5]. Microsecond laser was also used in SLM of 316 L and AlSi10Mg alloy to obtain higher process resolution [6,7], and IN718 alloy to address the serious element segregation and Laves phase formation problem [8]. Furthermore, a nanosecond laser was used in laser micro cladding of Ni-based composite coatings to improve the quality of coatings by controlling the heat affect zone [9]. These long- and short-pulse lasers certainly narrow the thermal field to some extent in powder bed, compared to a continuous laser, but the thermal effect is still difficult to control feasibly for the physical limitations of the laser-matter energy coupling mechanism.

A more effective heat source and precise heat control are promising for laser AM. One feasible route is to apply an ultrashort pulse laser as a heat source to process powders directly. Ultrashort-pulse lasers are usually defined as picosecond and femtosecond lasers, having features with spot sizes of several microns and lesser average power in comparison to continuous lasers [10]. These features are beneficial for controlling the formation of the molten pool, heat control, and consequently, mechanical properties.

Few studies have attempted to use ultrashort pulse laser in metal AM and made some progress. Sintered copper powder owing to the lack of formation of the molten pool was reported in the research of Kaden et al. where femtosecond laser at a repetition rate of 20 MHz were deployed [11]. Similar results, wherein there was no formation of a dense microstructure, were found in Liu’s investigation on Al-Li alloy AM using a femtosecond laser at a repetition rate of 20 MHz [12]. Ullsperger et al. investigated the impact of the pulse duration and pulse energy on the size of melt pool at a repetition rate of 1–20 MHz, they found the enhanced energy coupling efficiency in the keyhole melting mode using 500 fs at 20 MHz leads to a densification up to relative densities of 93 ± 1% [13].

Achieving full melting using a femtosecond laser has its physical limitations. In femtosecond pulses, only electrons are heated by the laser and the lattice remains cold [10]. The heat effect can be observed with increasing the repetition rate, because the femtosecond pulse duration was significantly lower than the characteristic heat diffusion time, the ablation mechanism remained dominant [14,15]. In addition, high repetition rate up to 20 MHz applied in previous studies mentioned above may bring up complex energy coupling mechanism. The shorter time intervals between consecutive pulses ensures enough heat accumulation but meanwhile decreases ablation threshold, which makes material removal more easily compared to lower repetition rates. In femtosecond pulses, high peak power density above a plasma threshold of 10^8^ W/cm^2^ combined with a high repetition rate up to a magnitude of MHz possibly cause plasma shielding effects that increase the process complexity in powder bed fusion [16,17].

Prolonging the laser pulse from femtosecond to picosecond shows the physical feasibility of full melting. The picosecond laser pulse duration is comparable to the time scale of the heat effect occurrence, including lattice heating and heat diffusion. Hence, the heat effect is a consequence of the laser-matter interaction at the picosecond timescale [18]. 

Compared with femtosecond lasers, the longer pulse duration of picosecond laser indicates that temperature rise and heat accumulation in the lattice could be accomplished under a sufficient number of pulses [19,20], demonstrating the picosecond laser’s potential as a heat source to achieve full melting and form a dense microstructure.

An essential prerequisite to obtaining a dense microstructure using a picosecond laser is the optimization of parameters and processing settings. Existing strategies for the application of ultrashort lasers to bulk material interaction have their limitations in supporting optimization, as gaps in the powder layer cause higher absorptivity, compared with bare plates [21,22]. For continuous laser–matter interaction research, previous studies on laser AM showed progress by real-time observation with in situ imaging technology and numerical simulation. For instance, in 2020, Zhao et al. [23] investigated the critical instability at moving keyhole tips, in PBF-L, using operando high-speed X-ray imaging, with a third-generation high-energy light source, at high spatial (2–3 μm/pixel) and temporal (0.1–7500 ns) resolutions. Khairallah et al. [24] established a high-fidelity multi-physics model to accurately capture laser–melt pool interactions on a meso-nanosecond scale. However, both real-time observation and numerical simulation have difficulties in tracking interactions on a temporal scale of picoseconds, not to mention the repetition rate effects.

In the present work, the aim was to obtain a complete melting and dense microstructure by using a picosecond pulse laser as a heat source at optimized process parameters in PBF-L. Lower repetition rate of 500 kHz was adopted to achieve full melting, compared to previous studies. The material used in this study was Ti6Al4V powder, and single-layer samples with certain thicknesses were prepared. The morphology and microstructure of the surface and cross-sections of the samples were studied systematically. The relationship between microstructure evolution in the cross section and attenuation of the heat effect in depth was illustrated. The mechanical properties of the samples were also investigated. The evidence of a dense melted pool with good mechanical properties obtained at optimal parameters indicates that the picosecond laser can be integrated as the heat source with the current micro-SLM to fabricate parts with accuracy control for the smaller size of the thermal filed.

## 2. Materials and Methods

### 2.1. Material

**Figure 1:**
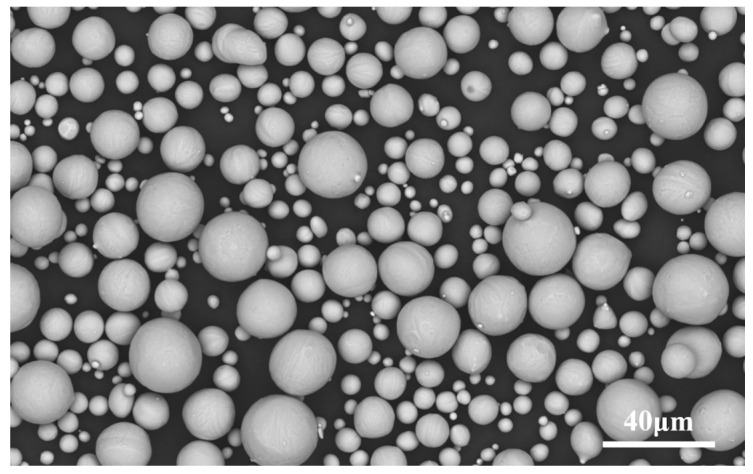
Morphology of Ti6Al4V powder under SEM.

Gas-atomized Ti6Al4V powder tailed for AM was used as the raw material (Avimetal Powder Metallurgy Technology Co., Ltd., Beijing, China). The powder morphology was observed using field-emission scanning electron microscopy (FE-SEM), and was found to be mainly spherical, as shown in Figure 1. The compositions and particle size distribution of the powders are listed in Table 1 and Table 2, respectively.

### 2.2. Equipment and Process

**Figure 2:**
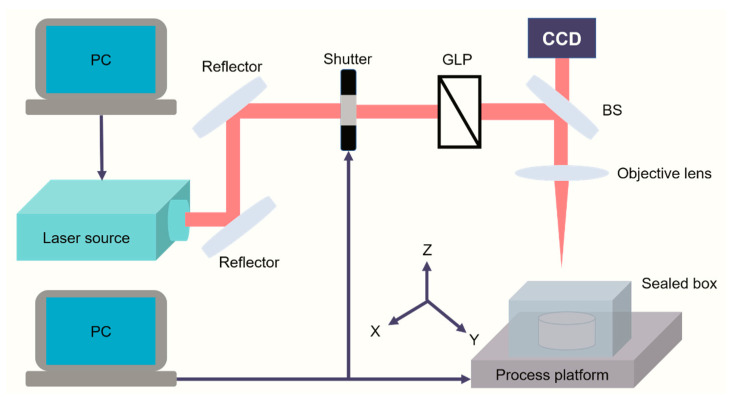
Schematic diagram of ultrashort pulse laser processing.

A commercially available titanium-doped sapphire ultrashort pulse laser (light conversion, Vilnius, Lithuania) was used for processing. The laser is capable of producing a maximum pulse energy of 4 mJ with a tunable pulse duration ranging from 200 fs to 10 ps and repetition rate between a single pulse and 1 MHz, at a wavelength of 1030 nm. The Ti6Al4V powder was filled in a cylindrical rubber container. A relatively flat surface of the powder bed was obtained after the excess powder was removed from the upper surface of the container using a scraper, thus, the plane where the powder bed was located and the upper surface of the container were almost in the same plane, to ensure the flatness of powder bed. The filled container in a customized optical glass sealed box was placed on a three-dimensional (3D) processing platform from Newport Corp. (Irvine, CA, USA), Laser μFAB^TM^, which possesses a movement resolution of 0.05 μm, maximum speed of 300 mm/s in the X-Y direction, and maximum movement of 5 mm in the Z direction. The laser beam was ultimately focused on the surface of the powder bed, as a spot of 36 μm diameter with a Gaussian profile, through an optical lens after passing through the delivery optics system. The laser spot remained stationary, as shown in Figure 2. The single tracks and muti-tracks with various hatch space settings were prepared to obtain optimal parameters. A raster scanning strategy was conducted by the movement of the process platform in the X-Y direction to print a batch of single-layer samples with sizes of 3 × 3 mm^2^.

### 2.3. Characterization

The as-printed tracks were observed using optical microscopy (OM). A batch of single-layer samples printed at optimal parameters were divided into two groups for characterization work. The surface morphology and microstructural evolution characteristics of the first group samples were observed using OM and FE-SEM (S-4800, Hitachi and Pro-X G6, Phenom). Part of the samples were also broken in half to investigate the cross-sectional microstructure using SEM (Pro-X G6, Phenom). The second group of samples, embedded in epoxy resin through grinding and polishing processes, was prepared for the subsequent nanoindentation characterization work. Instrumental indentation tests were performed using a triboindenter TI980 (Bruker, Billerica, MN, USA) to evaluate the hardness and elastic modulus of the single-layer Ti6Al4V alloy. A scanning probe microscope equipped on the machine was utilized to image the area for nanoindentation, and the peak load was 5 mN. In addition, X-ray computed tomography (X-CT) measurements were performed using the Xradia 500 Versa X-ray microscope at the maximum spatial resolution of 0.7 μm to examine the inner information of integral single-layer samples.

## 3. Results

### 3.1. Optimal Parameters for Dense Layer Fabrication 

**Figure 3:**
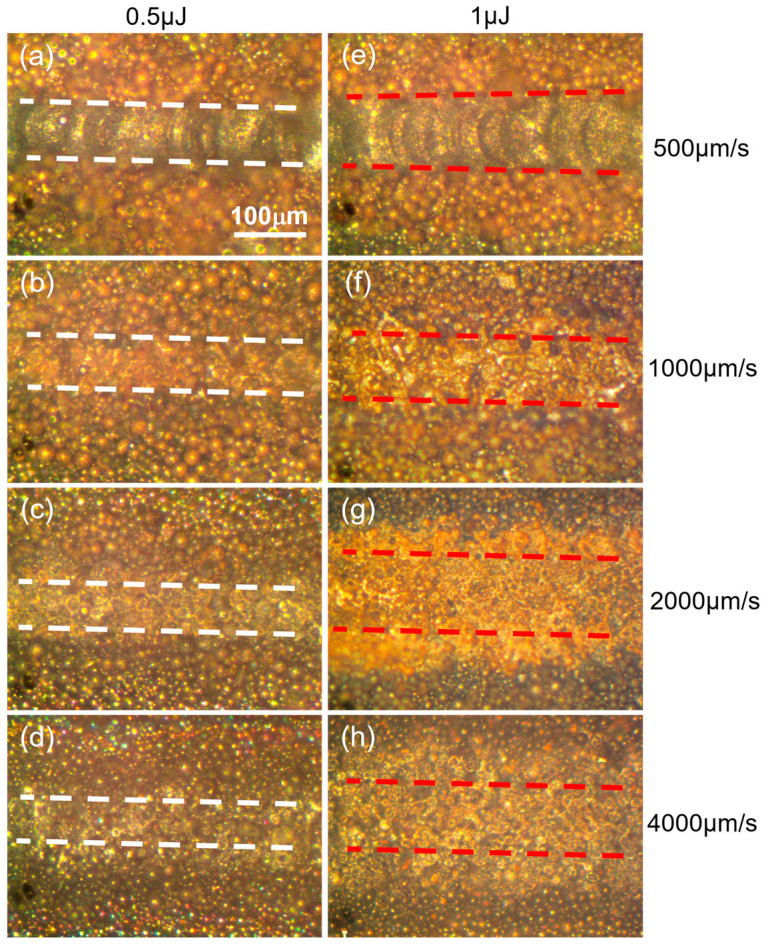
Effects of scanning velocity and pulse energy on the morphology revolution of single track at repetition of 500 kHz: (**a**–**d**) 0.5 μJ, (**e**–**h**) 1 μJ. All scale bars = 100 µm.

Figure 3 illustrates the impact of beam velocity on the densification behavior of track at pulse energy of 0.5 μJ and 1 μJ, respectively. The track morphology was highlighted by the white and red dash line under different parameters. Stable melting, partial melting, sintering and slight sintering occurred sequentially with increasing velocity from 500 µm/s to 4000 µm/s at two kinds of pulse energy. Fish-scale-like molten pools were produced at 500 µm/s as presented in Figure 3a,e where the width of track was roughly 60 µm and 100 µm at 0.5 μJ and 1 μJ, respectively. Compared with the morphology of track at 1 μJ, little discontinuity and some caters appeared in Figure 3a for less heat accumulation under the same amount pulses deposited in powder bed, causing molten pool instability. In this case, increasing pulse energy was beneficial to improve the molten pool stability, as long as it did not exceed the energy ablation threshold. Partial melting of powder was shown in Figure 3b,f in which no overlapped molten pools were observed though a wider range of partial melting zone was obtained at higher energy. With scanning velocity increasing from 1000 µm/s to 4000 µm/s, the melting morphology of powder both decreased significantly under two energy condition, shifting from melting to sintering state. 

The heat accumulation of ultrashort pulser laser in powder bed fusion additive manufacture process plays key role in realizing stable melting. In this study, heat accumulation, decided by the pulse energy and beam velocity at fixed repetition rate value, showed more sensitivity to scanning velocity, as the results of track revolution presented in Figure 3. Line energy density (LED), namely the product of pulse energy and repetition rate, divided by the beam velocity, was used to elaborate the heat accumulation preference. It can be calculated that the value of LED in Figure 3a,f was the same of 1 J/mm, others similar cases including Figure 3b,c,g,h where the value of LED was 0.5 J/mm and 0.25 J/mm, respectively. However, different melting behavior happened under the same LED, compared with the case of the decrease in pulse energy in half, double the velocity caused less heat accumulation, as a result of increasing in bema overlap rate, leading to less powder melting. Therefore, the densification of tracks showed more velocity reliance at fixed repetition rates. 

**Figure 4:**
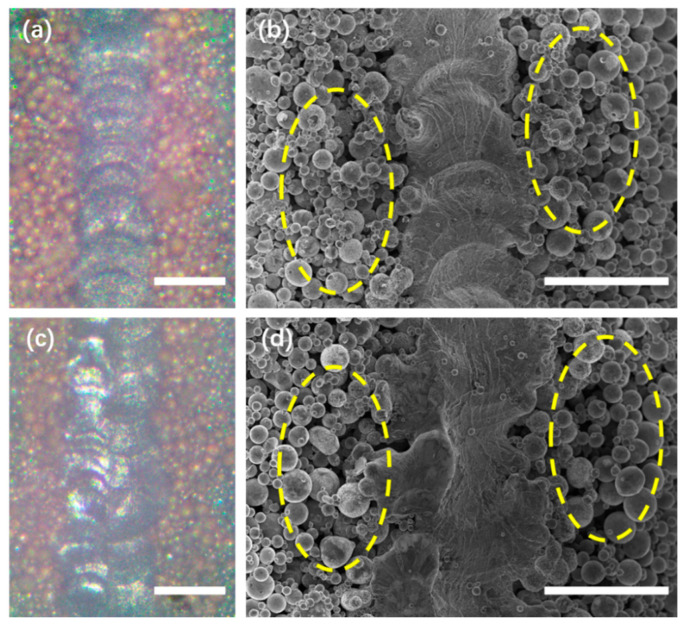
Morphology of single track and dual track under stable melting mode in Figure 3e: (**a**,**b**) Optical microscope and SEM image of a single track, (**c**,**d**) Optical microscope and SEM image of a pair up-down melted track where the hatch distance is 50 µm. The yellow dashed circles indicated the sintered powder at two sides of printed track. All scale bars = 100 µm.

Figure 4a shows the OM morphology of a single track at stable melting mode presented in Figure 3e. More details can be found in SEM image of Figure 4b where overlapped melted pools connected to form a continuous track, on two sides of which the powder sticked with each other to form a pre-sintered zone, highlighted in the yellow dash circle. Figure 4c shows the OM morphology of a pair up-down tracks at 50 μm hatch space where the newly formed track with 100 μm width at right converted its direction due to remelting relative to the previous track in the left. More details were shown in Figure 4d where no pores or micro-cracks were observed in the bonding zone, indicating that inter-track remelting ensured the densification process via picosecond laser source. In addition to the remelting of the previous solidified track, the intrinsic pre-sintering heat treatment applied before the following fusion process to the binding particles and making the powder bed rigid was beneficial to eliminate powder spatter and denudation, which is also observed in continuous mode laser PBF study [25]. Therefore, the pre-sintered zone circled out in Figure 4d also contributed to the densification in muti-track fabrication during the fusion process of raster scanning pattern. 

**Figure 5:**
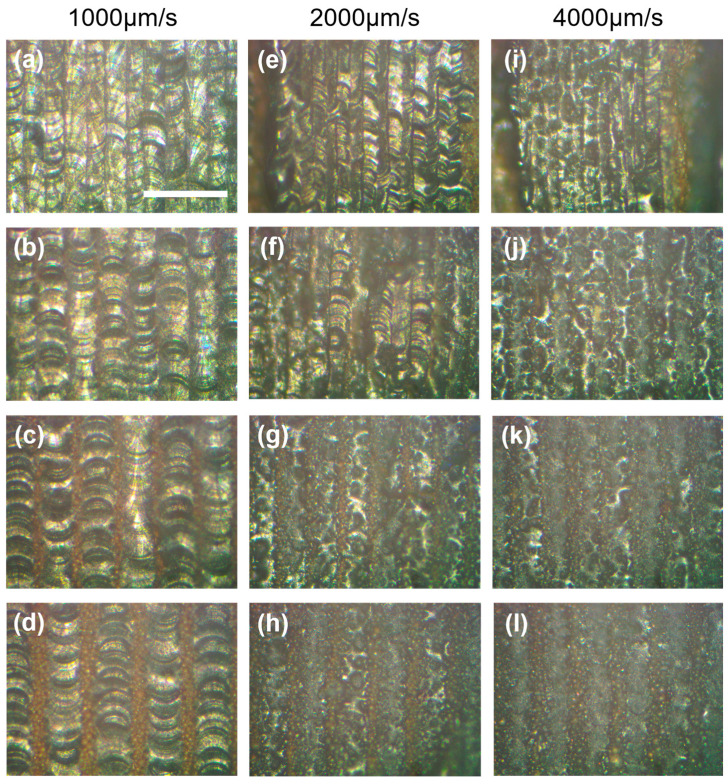
Effects of hatch space on the morphology revolution of single track and layer at 500 khz and 1 μJ under lack of energy mode: (**a**–**d**) 50 μm, 75 μm, 100 μm, and 125 μm at 1000 μm/s in Figure 3f, (**e**–**h**) 30 μm, 50 μm, 75 μm, and 100 μm at 2000 μm/s in Figure 3g, (**i**–**l**) 30 μm, 50 μm, 75 μm, and 100 μm at 4000 μm/s in Figure 3h. All scale bars = 200 µm.

To further explore the role of pre-sintering effect on the track formation, three lack of energy modes were used to print layers under various hatch space plans, respectively, the results are presented in Figure 5. Under partially melting mode, namely the track printed in Figure 3f, it is interesting to find that dense layer could be produced at 50 µm and 75 µm hatch distance, as shown in Figure 5a,b. With increasing in hatch distance from 75 µm to 125 µm, the failure of inter-track remelting appeared, leading to the lack of fusion, as shown in Figure 5c,d. However, the appearance of fish-scale-like track confirmed the positive effect of the sintered particles on the two sides of partially melting track, that is, converting the melting degree of mono track from partially melting to complete melting by enhancing the energy coupling. A similar phenomenon was also found under sintering mode, a dense layer could be still printed with relatively smaller hatch distance, as shown in Figure 5e,f. Balling tracks, instead of fish-scale-like track in Figure 5c, were shown in Figure 5g,h where the melting degree of tracks was improved significantly compared with the case of tracks in Figure 3g, further showing the advantage of pre-sintered zone. Under slightly sintering mode, the layer was still printed successfully at smallest hatch distance of 30 µm, though balling occurred with poor surface finish, as shown in Figure 5i. With increments in hatch distance, energy input per area decreased and partially melting and sintering occurred sequentially in Figure 5j–l, which means higher hatch distance was a limiting factor to these modes in muti-track printing. In summary, the energy coupling mechanism in muti-tracks printing shows obvious difference with that of in single track fabrication. From densification point of view, it is not necessary to print dense layer using lack of energy modes during multi-track printing for no obvious advantage of using these modes. However, the evidence of dense layer and melting degree elevation in muti-tracks fabrication under lack of energy modes shows the positive role of pre-sintering mechanism on track–track metallurgical bonding and densification.

**Figure 6:**
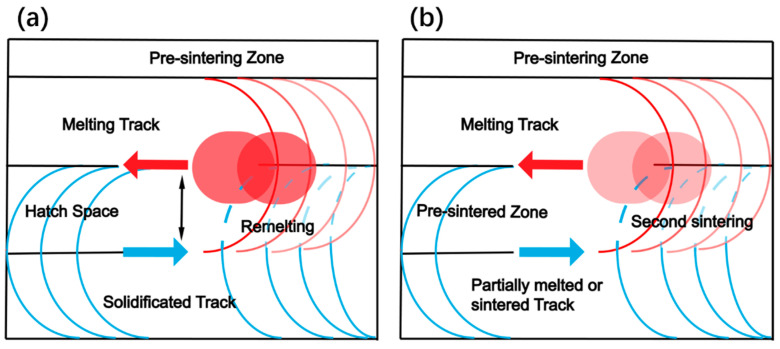
Schematic diagram of the formation mechanism of melted dense layer at two track formation modes: (**a**) stable melting mode, (**b**) lack of energy mode.

Figure 6 shows the schematic diagram of the formation mechanism of dense layer at stable melting and lack of energy mode at relatively low hatch space. Under stable melting mode in Figure 6a, the inter-track remelting dominates the whole fusion progress. The N + 1st track began to form, following which half of the Nth melted track was remelted. The orientation of the molten pool in the remelting area was reverse to the laser movement direction of the N + 1st track. It can be speculated that all intermediate tracks were remelted with the exception of the last track in muti-tracks fabrication. Under a lack of energy mode in Figure 6b, sintering mode for instance, second sintering during the N + 1st track scanning applied on the Nth sintered track is the reason for the melted pools formation. With the existence of sintered particles produced in the Nth scanning, the powder bed on the N + 1st track laser interplayed with was no longer loose particles but porous medium in sticked stage. The pre-sintered zone presented as a porous medium in the form of connected powders, which changes the laser powder energy coupling and improves the heat conduction and transfer behavior in the second sintering process, leading to the full melting of powder.

For printing dense layer sample fish-scale-like track was pronounced, and the two kinds of re-fusion modes were feasible. It is suggested that the parameters design also needs to take the pre-sintering phenomenon into account. To study the feature of layer sample, stable melting mode was selected in the following research and a set of optimal parameters listed in Table 3. 

### 3.2. Analysis of Surface of Single-Layer Samples

**Figure 7:**
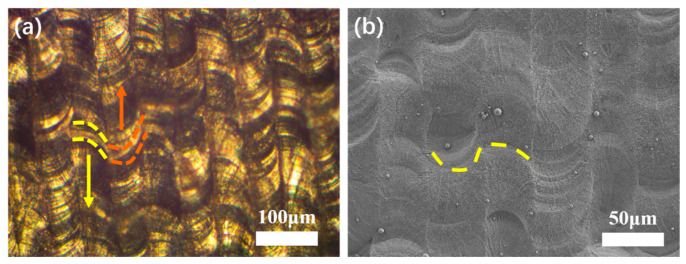
(**a**) Optical microscope image of the as-printed layer, (**b**) SEM image of the as-printed layer from top view.

The overlapped melted track morphology of a single-layer sample was observed in OM, as shown in Figure 7a. The overlapped molten pool is highlighted by the dashed line, and the arrows indicate the laser beam tracks on the powder bed along the scanning direction. The melted tracks confirmed the capability of the picosecond laser as a heat source, and the formation of a melting track under irradiation by an ultrashort pulse laser can be addressed from several perspectives as follows:

The laser energy density η, also called the fluence in ultrashort pulse lasers, must be below the ablation threshold. The threshold suggests whether the materials will undergo direct evaporation. Zheng proposed the ablation threshold of bulk Ti6Al4V at 0.1021 J/cm^2^ under a pulse duration of 10 ps using Equation (1) [26]. Herein, the laser energy density calculated using Equation (1) is 0.098 J/cm^2^, which is lower than the ablation threshold of bulk Ti6Al4V. Considering the intrinsic difference between bulk and powders, the small fluctuations in pulse energy and the uneven laser energy distribution in porous metallic powder, it was recommended that the fluence adopted was below the nominal ablative threshold of bulk metal.

(1) $$\eta =\frac{E_{single}}{\pi r^{2}}$$

where $E_{single}$ is pulse energy, and *r* is spot diameter of the laser beam.

The threshold calculated using Equation (1) disregards the difference between bulk and powder materials in the energy coupling mechanism of laser–matter interaction, and this should be carefully considered for the AM process. Zhang [27] studied the heat transfer in a thin powder layer subjected to a short-pulsed volumetric heat source. It was found that the decrease in the powder-layer surface temperature was a result of the increase in optical penetration depth, and consequently, the laser energy can penetrate deeper into the powder layer. Therefore, it is inferred that gaps in the powder bed with sufficient thickness play a key role in avoiding the powder ablation under picosecond irradiation. 

In addition, sustainable heat accumulation is a prerequisite for melting-pool formation. The repetition rate and heat source movement rate are two parameters that need to be optimized. Although the power density for ultrashort lasers can be extremely high, melting is only achieved under the condition of a sufficient number of pulses, as a single pulse of 10 ps exhibits insufficient energy. In this study, by increasing the repetition rate to 500 kHz with a fixed energy density, significant heat accumulation was realized, this kind of heat accumulation was similar to the previous studies [28]. The speed of the heat source movement was set at 500 μm/s to ensure the wettability of the molten pool at a rapid cooling rate.

Figure 7b shows SEM image of the surface microstructure of a single-layer sample consisting of fully dense tracks without sensible microcracks and pore defects. Some ridge-like structures can also be observed in Figure 7b, highlighted by the yellow dashed line. The orientation of the two adjoining ridges was the opposite, which was consistent with the direction of the laser movement between the two tracks. The formation of these ridges was likely related to the discontinuity of the pulse laser energy and fluid dynamics of the molten pool under extreme non-equilibrium conditions. It can be speculated that when the newly formed molten pool was connected to the previous one through wettability, with the spot moving ahead along the scan path, the end of the previous molten pool solidified for the rapid cooling rate induced by the short time interval between successive pulses. The front part of the molten pool still had sufficient wettability, so the ridge structure had a certain width. In addition, the melt pool instability caused by Marangoni convection may also have contributed to the protrusion and depression on the melted track [29].

### 3.3. Analysis of Cross Section of Single-Layer Samples 

**Figure 8:**
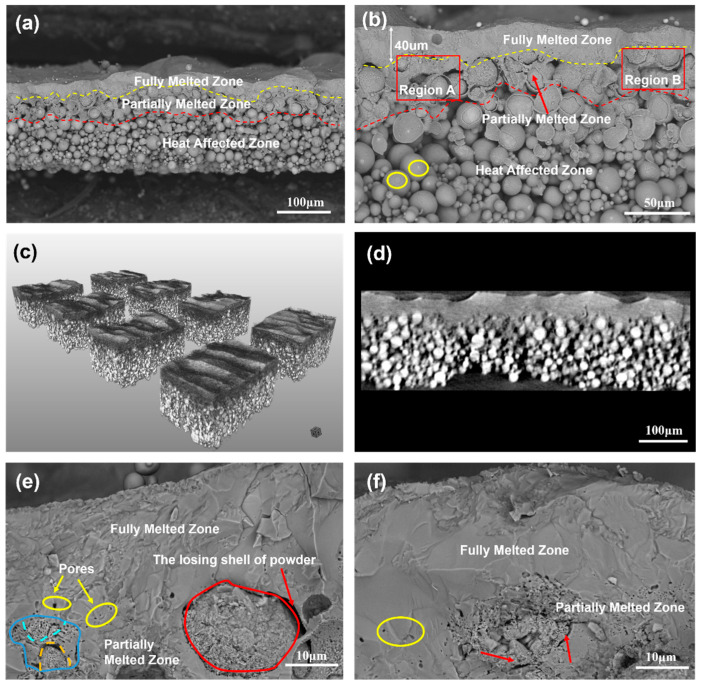
Characteristics of cross-sectional morphology: (**a**) SEM image of the overall cross-sectional morphology of the sample, (**b**) SEM image of local magnification of cross section, (**c**) A 3D XCT reconstruction of the single-layer sample within an observation volume of 740 μm × 740 μm × 180 μm after being divided into eight pieces, (**d**) A single XCT slice of the cross section in (**c**) Within an observation size of 740 μm × 180 μm, (**e**,**f**) Morphology of transition area of fully melted zone and partially melted zone.

The cross-sectional morphology of a single-layer Ti6Al4V sample with an average thickness of 200 μm was captured by FE-SEM in Figure 8a. The whole cross section with hierarchical structure is divided into three layers using colored dashed lines according to the shape of powders: fully melting zone (FMZ), partially melted zone (PMZ), and heat-affected zone (HAZ). More details on the morphological characteristics of the different regions are shown in Figure 8b.

The FMZ was lined with a dashed line with an average thickness of approximately 40 μm. The melted layer with a certain fluctuation thickness provides additional solid evidence supporting the formation of the molten pool, as discussed in Section 3.2. It is speculated that the spatters and the unsteady melted pool were responsible for the thickness fluctuation. Laser-induced spatters may have a shielding effect on the laser, leading to a lack of energy in the molten pool, thus causing a decrease in the melting depth [24]. In addition, thermal fluid dynamics such as convection or molten eruption may also contribute to the instability of the solidification process in the presence of oxides in the molten pool [30]. Some ridge-like structures that had been observed over the tracks from the top view (Figure 7b) were found in the background above the FMZ. Dense single layer Ni-based composite coatings with thickness of 10–15 μm at optimal parameters were also fabricated by laser micro cladding using nanosecond laser in study of Liu et.al where the power of 16 W, scanning speed of 20 mm/s and repetition rate of 20 kHz were adopted [9]. As to the fundamental of laser–metal energy coupling when laser irradiating metals, the physical processes and corresponding timescales include laser-electron heating (several hundred femtoseconds), electron-lattice heating (several picoseconds), melting, evaporation and re-solidification (several nanoseconds or longer) [10,18]. From the point of energy coupling scale, for nanosecond laser, the electron and lattice temperature had already reached equilibrium state during single 10ns pulse, while for a picosecond laser, it is not enough to accomplish the equilibrium process during single 10ps pulse width, showing a non-equilibrium state heat behavior. From the point of energy distribution, the time interval of two consecutive pulses in study of Liu was 50 μs, together with an LED of 0.8 J/mm, while a time interval of 2 μs together with 1 J/mm in this study generated much more molten volume. Therefore, a higher repetition rate of 500 kHz was needed to enhance the heat accumulation. Furthermore, in muti-layer building process of PBF, 40 μm thickness of Ti6Al4V layer can ensure the inter-layer remelting while the results of Liu et.al indicated that it seems to be only feasible in fabricating coatings instead of muti-layer AM when using nanosecond laser at low repetition rate. 

XCT was used to obtain high-resolution 3D images of the investigated single-layer samples, shedding light on whether all the powders had been fully melted in the FMZ. The XCT 3D reconstruction within an observation volume of 740 μm × 740 μm × 180 μm was divided into eight pieces, as shown in Figure 8c. The FMZ and HAZ were clear owing to the absorptivity difference of X-rays in dense and loose material, while the PMZ was the transition zone. More details can be observed in Figure 8d, where an XCT slice of the cross section was extracted with a size of 740 μm × 180 μm (width × height) to present the internal structure information of the FMZ. Under a spatial revolution of approximately 1 μm, there were no noticeable partial-melted powders, microcracks, or pores defects existing in the FMZ area. 

PMZ was formed between the yellow and red dashed lines, where partially melted powders were found near the bottom of the FMZ. The PMZ provides critical information to understand the heat transfer in the Z-axis direction and benefits further multilayer sample preparation. The depth of the PMZ was slightly lower than that of the FMZ. In region A of Figure 8b, this implies that the molten fluid in the FMZ penetrated into the deeper powder bed, and then the shell of the powders was heated to melt and subsequently connected with other powders as molten fluid. 

The transition zone between the FMZ and PMZ revealed the boundary of melting and the factors attributed to it. In Figure 8e, pores with sizes ranging from submicron to several microns were circled by yellow circles at the transition area of the FMZ and PMZ. Some melted powders were also found in the PMZ. A partially melted core–shell powder is lined with red circle at the lower right corner. The ditch between this particle and other particles was evidence of the absence of sufficient energy. Given that the D90 of the powder was 26.8 μm, the width of the ditch was approximately 3 μm, thus implying that the ditch may be attributed to the peeling of the powder shell. A similar case was also observed in Figure 8f where micro pores highlighted by yellow circle and partially melted core–shell powder highlighted by red arrow were observed. Apparently, the gap between the shell and core was responsible for inducing pores in the absence of sufficient energy. In addition, as shown in the lower left corner of Figure 8e, several partially melted powders with small particle sizes less than 10 μm were melted considerably. This indicates that powders with smaller particle sizes were easier to achieve complete melting because small powders have the advantage of reflection and scattering of the laser, thus promoting laser absorption. The size effect of powders on densification behavior in this study are in good agreement with those of Gu et al. [31]. It is further noted that the downward heat transfer in the deep powder bed may be affected by the larger size of the powder because of the limited heat diffusion depth. In region B of Figure 8b, two big powders in region B connected together through surface wetting, close to the bottom of the FMZ, showed little contribution to the heat transfer. The thickness of the FMZ over region B decreased significantly because of the two slightly melted powders. Sufficient melting penetration depth was hindered by the larger particle size powders, resulting in thickness fluctuation of the melted track. Therefore, the fraction of big size particles in the fine raw powder should be taken into consideration.

**Figure 9:**
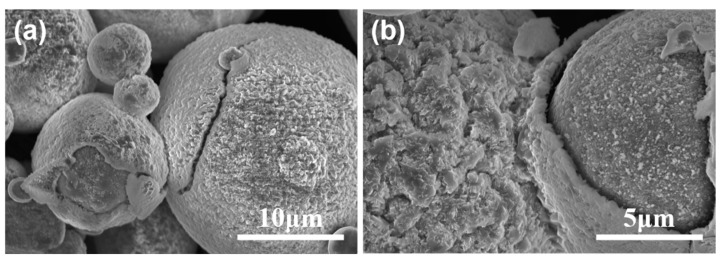
(**a**) SEM morphology of connected powder in HAZ, (**b**) SEM morphology of a neck formation of two powder via solid-phase sintering.

A heat-affected zone was formed below the red dashed lines where the main feature was that the powders were gently linked together, without scattering as a powder bed. Most powders remained spherical, signaling no melting in this zone. The connection of the powders in this zone may be attributed to a combination of force and heat. In Figure 8b, the plastic deformation induced by the force at the powder surface was circled out by yellow line. It is assumed that the recoil pressure generated by the plasma expansion on the powder layer and the shock wave increases the contact of powders, and this increases the contact between the powders and is conducive to heat transfer in the deep powder layer during a certain time [32,33]. The solid-phase sintering may also be attributed to the powder connection in HAZ, as shown in Figure 9a. More details were presented in Figure 9b where the binding phenomenon of the powders and the underlying evidence supporting the binding mechanism. Solid-state diffusion occurred at the contact point of the two particles, resulting in neck formation. Kruth [34] pointed out that this type of binding mechanism reflects solid-phase sintering, which explains how most of the powders in HAZ form an unbroken whole.

### 3.4. Thermal Behavior of Picosecond Laser in Powder Bed

**Figure 10:**
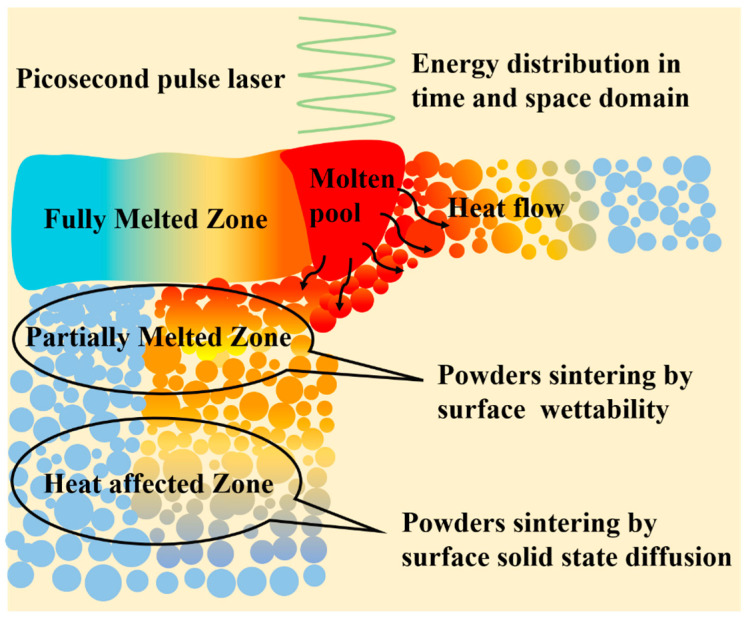
Schematic diagram of heat effect in powder bed after picosecond pulse laser energy deposited.

Figure 10 illustrates the heat effect generated in the powder bed after picosecond pulse laser radiation. The formation of FMZ relies on laser parameters, including laser density, repetition rate, and laser processing rate. In addition, gaps in the powder bed play a critical role in the FMZ. For a porous powder medium, the optical penetration depth is in the range of micrometers owing to the gaps in the powder layer [17,21]. In comparison, the penetration depth for bulk materials is normally on the scale of nanometers, as reported in previous studies on the material removal phenomenon and underlying ablation mechanism [35,36]. The penetration thickness at micrometers in picosecond laser irradiation leads to dissipation of light energy and attenuation within the powder layer. Consequently, melting disregards the discrete heat transfer mechanism in the powder bed [37]. The formation of partial melting in the PMZ and bonding in the HAZ is still unclear, but the selection of particle size is another effective factor. In this study, the partial melting and solid-phase diffusion may have a direct relationship with the drastic variation in the local absorptance determined by the particle volume fraction [38].

To study the energy efficiency (considering the case of forming molten pool under stable melting mode) of picosecond pulse laser-powder interaction, herein, a semi-quantitative model is proposed to make an estimation on the energy efficiency λ of the picosecond laser, as shown in Equation (2).

(2) $$\lambda =\frac{E_{heat}}{\alpha E_{input}}\approx \frac{C\rho V_{s}\left(T_{1}-T_{2}\right)+\rho V_{s}Q_{lh}}{\alpha \times E_{single}\times t_{s}\times R}$$

where $E_{heat}$ denotes the total heat required for complete melting of powders and latent heat release within a nominal volume, $E_{input}$ denotes the total heat input by the pulse laser within a specific time, *α* is the energy loss coefficient, the first term of the molecule on the right side of Equation (2) denotes the volumetric melting enthalpy [39] and $Q_{lh}$ in the second term denotes the latent heat of fusion, *C* is the specific heat capacity of the powders, ρ is the density of the loose powder, $V_{s}$ is the characteristic volume of the fully melted zone, $T_{1}$ denotes the melting point of the powders, $T_{2}$ denotes the temperature of the pre-irradiation powders, $E_{single}$ is the single pulse energy, $t_{s}$ denotes the characteristic time required for the laser to travel a certain distance, and R is the repetition rate.

A geometric model was defined with the characteristic size extracted from the fully melted zone. The width of the melted pool was close to 100 μm, and the depth of the fully melted zone was presumed to be 40 μm. A simplified estimation was set according to the following: the laser movement on the powder surface distance of 100 μm, and $t_{s}$ assigned a value of 0.2 s. From Equation (2) the total heat input by the pulse laser $E_{input}$ is calculated to be 0.1 J within 0.2 s. 

To continue this semi-quantitative study, some assumptions and simplifications are required. The geometric model was assumed to be bulk Ti6Al4V, and the plasma or nanoparticles evaporation shielding effects were ignored. α is assigned a value of 0.5, considering the reflection of powder to the laser pulses. The data of thermophysical properties of Ti6Al4V were obtained from a previous study [40,41] where *C*, ρ, $T_{1}$ and $Q_{L}$ equals 700 J/(kg·K), 4000 kg/m^3^, 1903 K, and 286 kJ/kg, respectively.

In this way, the $E_{heat}$ was calculated from Equation (2) to yield 0.0023 J approximately. Hence, the estimated energy efficiency of the picosecond pulsed laser in this study was close to 4.5%. Considering the variation in different USP machines, the value of estimated thermal efficiency can provide reference for the parameter choice in similar metal powder bed fusion via USP. In continuous laser of SLM, the process efficiency is typically 2–20%, as concluded in study of Mishra et al. [42]. For instance, the value of process efficiency of AlSi10Mg with SLM in study of Leis et.al was 7–8% [43]. The estimated energy efficiency of 4.5% using picosecond laser was relatively low in comparison with that of continuous laser mode.

### 3.5. Mechanical Analysis

**Figure 11:**
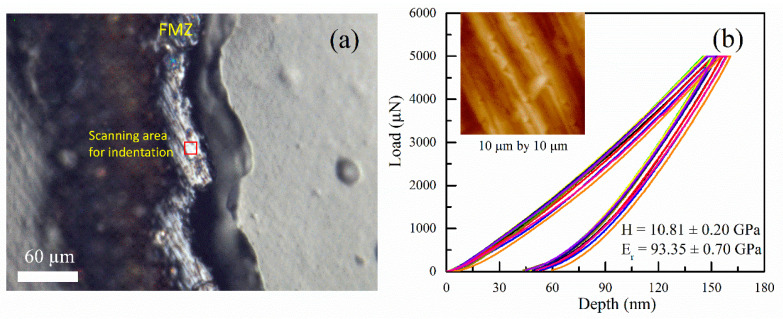
Cross-section morphology of single-layer Ti6Al4V sample via optical microscope (**a**). Red square in the FMZ shows the region where indentation tests were performed, and the hardness and elastic modulus values are calculated from the load–displacement curves (**b**), residue indents after indentation tests can be observed in the scanning image (inset of (**b**)).

The hardness and elastic modulus of the cross-sectional surface of the FMZ were measured to evaluate the quality of powder melting of the Ti6Al4V alloy. As displayed by OM in Figure 11a, FMZ shows a rough surface with polish scratches, and the area in the red square was further scanned for nano-indentation tests. The load–displacement curves for the indentation tests are plotted in Figure 11b. The inset of Figure 11b shows the scanning image of the FMZ surface after the tests. Over 15 indentation tests were performed on a relatively flat region to minimize the effect of the rough surface. According to Oliver-Pharr method [44], the hardness H and reduced modulus E_r_ values are calculated to be 10.81 ± 0.20 GPa and 93.35 ± 0.70 GPa, respectively. The high hardness of the FMZ probably results from the nanostructured Ti alloy after the laser-assisted remelting process [45]. Taking the elastic properties of the diamond indenter (*E_i_* = 1141 GPa, *ν_i_* = 0.07) [46], and the Poisson’s ratio of Ti6Al4V alloy to be 0.342 [47] and inputting these into the relation [48]:$\;1/E_{r}=\left(1-\nu_{i}^{2}\right)/E_{i}+\left(1-\nu_{s}^{2}\right)/E_{s}$, the elastic modulus of the Ti6Al4V alloy in the FMZ was calculated to be 89.74 ± 0.74 GPa. This value is slightly lower than that of wrought Ti6Al4V alloy (113.1 GPa) [49]. However, it agrees well with the same alloy fabricated using selective laser melting (SLM) in which the value ranged from 91 Gpa to 109 Gpa [48], thus indicating that the Ti6Al4V fabricated by picosecond laser is rather dense and exhibits significant strength.

From the point of application view, the existence of dense molten pool with fine size and mechanical properties suggests the potential and advantages in the fabrication of microstructure parts via micro-LPBF (μ-LPBF) technology by using picosecond laser. Compared with traditional L-PBF, the feature of μ-LPBF is that the beam size is no more than 40 µm, powder size no more than 20 µm and layer thickness around 10 µm, thus high printing resolution is obtained [50,51]. Therefore, the process feature and results of this study imply that picosecond laser could be a feasible and desirable heat source in metal micro-SLM.

## 4. Conclusions

In this study, dense single layer Ti6Al4V samples were fabricated via picosecond pulse laser basing on PBF-L for the first time, the heat effect was investigated during the interaction of picosecond pulse laser with Ti6Al4V powder. The main conclusions drawn are summarized as follows:

The track and layer formation mechanism were discussed, and a set of optimal parameters were obtained to printed fish-scale-like Ti6Al4V layer sample where the repetition rate of 500 kHz, pulse energy of 1 μJ and scanning velocity of 500 μm/s were adopted, ensuring enough heat accumulation in realizing the complete melting of powder and stability of molten pools. The dense layer was resulted from the inter-track remelting and pre-sintering mechanism.

Highly dense microstructure of layer sample detected by SEM and X-CT were manufactured using a picosecond laser heat source. The elastic modulus of the printed dense layer tested by nano-indentation was 89.74 ± 0.74 GPa.

The cross-sectional microstructure has a three-zones structure from top to bottom in the sequence of the fully melted zone with average thickness of 40 μm, the partially melted zone, and the heat-affected zone. The fine feature size and limited thermal filed demonstrates the potential for heat control in micro-LPBF.

## Figures and Tables

**Table 1:** Chemical compositions of Ti6Al4V powder(wt%).

Ti	Al	V	C	Fe	O
Bal	5.76	4.07	0.05	0.08	0.18

**Table 2:** Particle size distribution of Ti6Al4V powder.

D_10_	D_50_	D_90_
7.89 μm	15.7 μm	26.8 μm

**Table 3:** A set of optimal parameters used for fabricating single-layer Ti6Al4V samples via picosecond pulse laser.

Laser Parameters	Value
Wavelength	1030 nm
Pulse Energy	1 μJ
Pulse duration	10 ps
Platform velocity	500 μm/s
Repetition rate	500 kHz
Hatch space	50 μm
Spot diameter	36 μm

## Data Availability

The data presented in this study are available on request from the corresponding author.
